# Prevalence of the EH1 Groucho interaction motif in the metazoan Fox family of transcriptional regulators

**DOI:** 10.1186/1471-2164-8-201

**Published:** 2007-06-28

**Authors:** Sergey Yaklichkin, Alexander Vekker, Steven Stayrook, Mitchell Lewis, Daniel S Kessler

**Affiliations:** 1 Department of Cell and Developmental Biology, University of Pennsylvania School of Medicine, 1110 Biomedical Research Building II/III, 421 Curie Boulevard, Philadelphia, PA 19104, USA; 2 Department of Economics, University of Pennsylvania, 328 McNeil Building, 3718 Locust Walk, Philadelphia, PA 19104, USA; 3 Department of Biochemistry and Biophysics, University of Pennsylvania School of Medicine, 813B Stellar-Chance Laboratories, 422 Curie Boulevard, Philadelphia, PA 19104, USA

## Abstract

**Background:**

The Fox gene family comprises a large and functionally diverse group of *forkhead*-related transcriptional regulators, many of which are essential for metazoan embryogenesis and physiology. Defining conserved functional domains that mediate the transcriptional activity of Fox proteins will contribute to a comprehensive understanding of the biological function of Fox family genes.

**Results:**

Systematic analysis of 458 protein sequences of the metazoan Fox family was performed to identify the presence of the engrailed homology-1 motif (eh1), a motif known to mediate physical interaction with transcriptional corepressors of the TLE/Groucho family. Greater than 50% of Fox proteins contain sequences with high similarity to the eh1 motif, including ten of the nineteen Fox subclasses (A, B, C, D, E, G, H, I, L, and Q) and Fox proteins of early divergent species such as marine sponge. The eh1 motif is not detected in Fox proteins of the F, J, K, M, N, O, P, R and S subclasses, or in yeast Fox proteins. The eh1-like motifs are positioned C-terminal to the winged helix DNA-binding domain in all subclasses except for FoxG proteins, which have an N-terminal motif. Two similar eh1-like motifs are found in the zebrafish FoxQ1 and in FoxG proteins of sea urchin and amphioxus. The identification of eh1-like motifs by manual sequence alignment was validated by statistical analyses of the Swiss protein database, confirming a high frequency of occurrence of eh1-like sequences in Fox family proteins. Structural predictions suggest that the majority of identified eh1-like motifs are short α-helices, and wheel modeling revealed an amphipathicity that supports this secondary structure prediction.

**Conclusion:**

A search for eh1 Groucho interaction motifs in the Fox gene family has identified eh1-like sequences in greater than 50% of Fox proteins. The results predict a physical and functional interaction of TLE/Groucho corepressors with many members of the Fox family of transcriptional regulators. Given the functional importance of the eh1 motif in transcriptional regulation, our annotation of this motif in the Fox gene family will facilitate further study of the diverse transcriptional and regulatory roles of Fox family proteins.

## Background

DNA-binding transcriptional regulatory proteins have a modular structure and are composed of a sequence-specific DNA-binding domain and trans-regulatory domains. Multiple studies have shown that short conserved peptide regions mediate the biological functions of trans-regulatory domains. In the case of transcriptional repressors, such short protein regions can autonomously mediate repression when fused to a heterologous DNA-binding domain [1,2]. It appears that these conserved regions form either α-helices or binding pockets to provide specific interacting surfaces for transcriptional corepressors. For instance, the Sin3 interaction motif of NRSF/REST adopts a short amphipathic α-helix that mediates specific physical interactions with the Sin3 transcriptional corepressor [3]. In the present study, we focus on identifying and analyzing the Engrailed homology region-1 (eh1) transcriptional repression motif in the Fox gene family of *forkhead*-related transcriptional regulators. This motif is known to mediate specific physical interactions of a number of protein families with transcriptional corepressors of the TLE/Groucho protein family [4-7].

The eh1 motif is composed of eight amino acid residues with the sequence pattern FS(I/V)XXΦΦX, with X representing any non-polar or charged residue and Φ representing branched hydrophobic residues. The eh1 motif was originally identified as a conserved N-terminal sequence shared between the *Drosophila *Engrailed protein and its vertebrate orthologs [6]. Functional analysis of the Engrailed protein has shown that the eh1 motif is required for active transcriptional repression in vivo, as well as for the physical interaction with Groucho corepressors [7,8]. An eh1-like motif was also identified in eight classes of the homeodomain protein superfamily (Emx, Dlx, Gsc, Hex, Msh, Six, Oct and Vnd) [5,9,10]. Further in vivo and in vitro studies have shown that the eh1-like motif of Gsc, Nkx, Hex and Six is required for repression function in vivo by recruiting the TLE/Groucho corepressors [5,9,11].

Eh1-like motifs have also been found in several members of the Fox family of *forkhead*-related transcriptional regulators [12]. Fox proteins are essential transcriptional regulators of embryogenesis, homeostasis, metabolism, and aging in metazoan organisms [13]. The highly conserved DNA-binding domain of Fox family proteins is characterized by the formation of three α-helixes, three β-strands and two loops resembling wings [14], thus the winged helix DNA-binding domain (WHD) designation. The WHD is flanked by N- and C-terminal regions that share low similarity among the Fox protein subclasses. The initial classification of Fox proteins based on sequence-relatedness within the WHD established fifteen subclasses of the Fox gene family [15], and four additional Fox subclasses were subsequently identified [16,17]. An updated list of Fox gene family members is available online [18].

Sequence analysis of several Fox proteins revealed that a short conserved C-terminal region of FoxA proteins (conserved region II or CII) was similar to the eh1 motif [12]. Further biochemical studies showed that FoxA2 physically interacts with TLE1, a mammalian Groucho protein, via the CII region [19]. These data suggest that the CII region not only resembles the eh1 motif in sequence, but also in the ability to directly binding Groucho/TLE corepressors. In addition, the *Drosophila *FoxG ortholog, Slp1, physically interacts with Groucho via an N-terminal eh1-like motif [20]. Furthermore, our recent studies in *Xenopus *have shown that FoxD3 can associate with the *Xenopus *Groucho ortholog, Grg4, via an eh1-like motif. The FoxD3 eh1 motif is essential for a functional interaction with Grg4 and for transcriptional repression in vivo [21]. These observations suggest an interaction of Groucho corepressors with multiple Fox family proteins, and prompted us to systematically examine all subclasses of the Fox gene family for the presence of eh1-like motifs. Given the functional importance of the eh1 motif in transcriptional regulation, annotation of the presence, pattern of distribution, and structural characteristics of this motif in the Fox gene family will facilitate further study of the diverse transcriptional and regulatory roles of Fox family proteins.

Here, we present a complete systematic analysis of the presence of eh1-like motifs in metazoan Fox proteins. Eh1-like motifs are identified in more than 50% of Fox proteins representing ten Fox family subclasses (A, B, C, D, G, E, H, I, L and Q) and statistical analyses of the Swiss protein database confirm a frequent occurrence of the motif in the Fox family. Secondary structure analysis of these Fox proteins predicts that the eh1-like motifs adopt a short amphipathic α-helical structure. Taken together, the results point to a functional interaction of TLE/Groucho corepressors with many members of the Fox family and identify structural features of the eh1 motifs that will facilitate further study of the physical interaction of Fox proteins with TLE/Groucho corepressors.

## Results

### Identification of eh1-like motifs in ten subclasses of the Fox gene family

We performed a systematic analysis of 458 yeast and metazoan protein sequences belonging to nineteen subclasses of the Fox family of transcriptional factors for the presence of eh1-like motifs. An initial manual search was conducted for the presence of sequences composed of eight amino acids with a highly conserved hydrophobic core matching the eh1 motif pattern of FSΦXXΦΦX (X, non-polar or charged residue; Φ, branched hydrophobic residue). Conserved regions of aligned orthologous Fox protein sequences were examined for homology to the eh1 consensus sequence. Eh1-like motifs were identified in Fox protein sequences of 10 subclasses, including the A, B, C, D, E, G, H, I, L and Q, but not in Fox proteins of the F, J, K, M, N, O, P, R and S subclasses (Table 1). Fox proteins containing an eh1-like motif were found across multiple animal phyla, and included chordates, hemichordates, and a variety of invertebrates, but not yeast (Tables 2 and 3). The identified motifs exhibit high similarity to the *Drosophila *eh1 motif in the range of 50–87%. To summarize the results, a phylogenetic tree for the Fox gene family was constructed in which the presence of an eh1-like motif within individual Fox proteins is indicated [see Additional files 1 and 2].

**Table 1:** Occurrence of eh1-like motifs in the Fox subclasses.

**Fox subclass**	**Total number of proteins^a^**	**Number of eh1-postive proteins^b^**	**Number of eh1-negative proteins**
A	39	37	2
B	40	40	0
C	27	24	3
D	74	55	19
E	22	15	7
F	19	0	19
G	21	21	0
H	14	11	3
I	25	5	20
J	29	0	29
K	15	0	15
L	21	6	15
M	9	0	9
N	26	0	26
O	8	0	8
P	25	0	25
Q	26	26	0
R	13	0	13
S	5	0	5

^a ^Number of proteins from each Fox subclass analyzed for the presence of an eh1-like motif.

^b ^Proteins containing a sequence with at least 50% similarity to the eh1 motif of the *Drosophila *engrailed homeodomain protein [7].

**Table 2:** List of the identified eh1-like motifs in eight subclasses of invertebrate Fox proteins.

**Subclass**	**Protein**	**Motifs^a^**	**Homology to eh1 motif^b^**	**Position^c^**	**Protein length**	**Species**	**Accession number**
A							
	FoxA	**FAIKNII**A	62.5%	243–250	321	*H. vulgaris*	AAO92606
	FoxA	**FAIKNII**A	62.5%	215–222	286	*N. vectensis*	42374841
	FoxA	**FSIDRIM**H	50%	412–419	485	*D. japonica*	9309317
	FoxA	**FSITRLL**P	75%	302–309	350	*H. armigera*	57791692
	FoxA	**FSITNLM**S	62.5%	375–382	435	*P. vulgata*	22859616
	FoxA	**FSITRLL**P	62.5%	300–307	349	*B. mori*	112983681
	FoxA	**FSITRLL**P	62.5%	372–379	435	*A. aegypti*	108881332
	FoxA	**FSITRLL**P	62.5%	374–381	437	*A. gambiae*	55233684
	FoxA	**FSINRLL**P	62.5%	452–459	510	*D. melangaster*	7301684
	FoxA	**FSINRLL**P	62.5%	370–377	431	*T. castaneum*	86515352
	FoxA	**FSITRLL**P	75%	460–467	570	*A. mellifera*	110759792
	FoxA	**FSINSII**P	62.5%	377–384	440	*S. purpuratus*	91983614
	FoxA5	**FSISSLM**N	62.5%	452–459	587	*C. intestinalis*	AAB61227
	FoxA5	**FSISNLM**S	87.5%	342–349	403	*B. floridae*	CAA65368
	FoxA5	**FSISSLM**N	62.5%	441–448	567	*M. oculata*	AAB69278
B							
	FoxB	**FAIENLI**G	62.5%	151–158	262	*N. vectensis*	ABA03229
	FoxB	**FSIESIL**S	75%	229–236	237	*C. elegans*	AAA28104.1
	FoxB	**FTIESLI**T	75%	222–229	372	*D. melangaster*	17977684
	FoxB	**FTIESLI**T	75%	172–179	241	*T. castaneum*	91082601
	FoxB	**FTIESLI**T	75%	189–196	198	*A. gambiae*	EAA07672
	FoxB	**FTIENII**A	75%	313–320	365	*A. mellifera*	110759134
	FoxB	**FTIENII**S	87.5%	187–194	360	*S. purpuratus*	NP999797
	FoxB	**FSIENII**S	87.5%	305–312	475	*C. intestinalis*	CAD58964
	FoxB	**FNIENII**A	62.5%	181–188	289	*B. floridae*	CAD44627
C							
	FoxC	**FTVDSLM**N	50%	260–267	508	*D. melangaster*	17975538
	FoxC	**FTVDSLM**N	50%	266–273	496	*A. gambiae*	EAA11069
	FoxC	**FTVDSLM**N	50%	251–258	412	*A. aegypti*	108876322
	FoxC	**FSVDALM**N	50%	304–311	495	*A. mellifera*	110758357
	FoxC	**YTVDSLM**A	50%	258–265	479	*S. purpuratus*	72007114
	FoxC	**FSVDNIM**T	75%	233–300	497	*B. floridae*	57337372
D							
	FoxD	**FMISNLL**K	75%	434–441	444	*S. domuncula*	CAE51209
	FoxD	**FSMESIL**S	62.5%	3–10	333	*C. elegans*	17536629
	FoxD	**FSISHII**S	87.5%	393–400	455	*D. japonica*	BAC10918
	FoxD	**FRIETLI**G	50%	435–442	456	*D. melangaster*	17647421
	FoxD	**FSIENLI**G	75%	491–498	504	*A. aegypti*	10886922
	FoxD	**FSIDALI**G	62.5%	313–320	354	*A. mellifera*	110759337
	FoxD	**FTIDSLL**N	62.5%	308–315	401	*S. purpuratus*	115953031
	FoxD	**FSIESLI**G	62.5%	377–384	506	*C. savignyi*	BAB68347
	FoxD	**FSIENII**G	75%	311–318	402	*B. floridae*	AF512537
E							
	FoxE	**FSIENII**G	75%	207–214	393	*C. intestinalis*	BAC57420
	FoxE4	**FSIDNII**A	75%	227–234	381	*B. floridae*	18653452
G							
	FoxG	**FSIENIL**K	75%	12–19	318	*M. leidyi*	AAN17798
	FoxG	**FSIRQML**D	50%	16–23	260	*D. japonica*	BAC10917
	FoxG	**FSILDLC**P	37.5%	4–11	270	*C. elegans*	17569837
	FoxG	**FSINSIL**P	50%	18–25	424	*A. gambiae*	EAA43390
	FoxG	**FGMDRLL**G	37.5%	284–291	424	*A. gambiae*	EAA43390
	FoxG	**FSISSIL**P	75%	156–163	444	*T. castaneum*	91080905
	FoxG	**FNMERLL**A	37.5%	381–388	444	*T. castaneum*	91080905
	FoxG1	**FSIRSIL**P	62.5%	51–58	451	*A. mellifera*	110756018
	FoxG1	**FSMERLL**Q	37.5%	328–335	451	*A. mellifera*	110756018
	FoxG1	**FSIDAIL**A	62.5%	12–19	322	*D. melangaster*	CAA46890
	FoxG2	**FSIDAIL**P	62.5%	62–69	445	*D. melangaster*	CAA46891.1
	FoxG	**FSVESML**S	62.5%	34–41	507	*S. purpuratus*	72179617
	FoxG	**FSVERLL**S	75%	396–403	507	*S. purpuratus*	72179617
	FoxG1	**FSIRRML**S	62.5%	20–27	402	*B. floridae*	AF067203
	FoxG1	**FSVERLL**S	75%	286–293	402	*B. floridae*	AF067203
L							
	FoxL1	**FTIDNII**G	75%	356–363	365	*D. melangaster*	Q02360
	FoxL1	**FSIDNIL**A	75%	299–306	521	*S. purpuratus*	72009133
Q							
	FoxQ1	**FSIDSIL**G	62.5%	251–258	408	*S. purpuratus*	82706210
	FoxQ1	**FSIESIL**S	75%	268–275	385	*C. intestinalis*	70569660
	FoxQ1	**FSIDAIL**S	75%	226–233	324	*B. floridae*	CAH55831
	FoxQ2b	**FDVESLL**R	50%	282–289	380	*C. hemisphaerica*	108796163
	FoxQ2a	**FSIENIL**G	75%	325–332	387	*C. hemisphaerica*	108796161
	FoxQ2	**FTIEAIL**E	62.5%	221–228	230	*C. elegans*	17505695
	FoxQ2	**FDVASLL**A	50%	348–355	599	*D. melangaster*	66571262
	FoxQ2	**FDVASLL**A	50%	233–240	299	*T. castaneum*	91076112
	FoxQ2	**FDVESLL**R	50%	232–239	307	*A. gambiae*	XP566358
	FoxQ2	**FSIENLA**Q	62.5%	4–11	329	*S. purpuratus*	ABB89473
	FoxQ2	**FSIDRLV**G	62.5%	4–11	271	*B. floridae*	AY163864
Orphans							
	Fox1	**FRIEFLL**K	50%	276–283	285	*N. vectensis*	ABA03228
	Fox1	**FSISKLI**L	75%	211–218	218	*S. domuncula*	CAE51213

^a ^The highly conserved core of the eh1-like motifs are indicated in bold.

^b ^The percent similarity between the identified Fox eh1-like motifs and the eh1 motif (FSISNILS) of the *Drosophila *engrailed homeodomain protein [7].

^c ^The location of the motifs within the amino acid sequence of the individual Fox proteins.

**Table 3:** List of the identified eh1-like motifs in ten subclasses of chordate Fox proteins.

**Subclass**	**Protein**	**Motif^a^**	**Homology to eh1 motif^b^**	**Position^c^**	**Protein length**	**Species**	**Accession number**
A							
	FoxA1	**FSINNLM**S	75%	359–366	427	*D. rerio*	AAH65668
	FoxA1a	**FSINNLM**S	75%	356–363	428	*X. laevis*	AAN76331
	FoxA1b	**FSINNLM**S	75%	355–362	427	*X. laevis*	AAA17050
	FoxA1	**FSINNLM**S	75%	394–401	466	*R. norvegicus*	6981034
	FoxA1	**FSINNLM**S	75%	396–403	468	*M. musculus*	P35582
	FoxA1	**FSINNLM**S	75%	400–407	472	*H. sapiens*	24497501
	FoxA2	**FSINNLM**S	75%	342–349	409	*D. rerio*	18858687
	FoxA2a	**FSINNLM**S	75%	351–358	434	*X. laevis*	45361699
	FoxA2	**FSINNLM**S	75%	354–361	438	*G. gallus*	NP990101
	FoxA2	**FSINNLM**S	75%	377–384	459	*M. musculus*	6753898
	FoxA2	**FSINNLM**S	75%	376–383	458	*R. norvegicus*	NP036875
	FoxA2	**FSINNLM**S	75%	376–383	457	*H. sapiens*	24497504
	FoxA3	**FSITNLM**S	87.5%	376–383	441	*D. rerio*	18858689
	FoxA3	**FSITNLM**S	87.5%	259–266	324	*S. salar*	AAC16333
	FoxA3	**FSINNLM**S	75%	307–314	353	*M. musculus*	22477526
	FoxA3	**FSINNLM**S	75%	394–401	466	*R. norvegicus*	CAA39418.1
	FoxA3	**FSINNLM**S	75%	304–311	350	*H. sapiens*	24497506
	FoxA4	**FSITNLM**S	87.5%	345–352	417	*A. mexicanum*	AAC60128
	FoxA4a	**FSITQLM**S	75%	328–335	399	*X. laevis*	CAA46290
	FoxA4b	**FSITQLM**S	75%	328–335	400	*X. laevis*	AAB22027
	FoxA5	**FSISSLM**N	62.5%	452–459	587	*C. intestinalis*	AAB61227
	FoxA5	**FSISNLM**S	87.5%	342–349	403	*B. floridae*	CAA65368
	FoxA5	**FSISSLM**N	62.5%	441–448	567	*M. oculata*	AAB69278
B							
	FoxB	**FSIENII**S	87.5%	305–312	475	*C. intestinalis*	CAD58964
	FoxB	**FNIENII**A	62.5%	181–188	289	*B. floridae*	CAD44627
	FoxB1	**FAIENII**A	62.5%	164–171	297	*D. rerio*	AAH56754
	FoxB1	**FAIESII**A	62.5%	171–178	289	*T. nigroviridis*	47209343
	FoxB1	**FAIENII**A	62.5%	167–174	319	*X. laevis*	AAC62623
	FoxB1a	**FAIENII**A	62.5%	170–177	325	*M. musculus*	Q64732
	FoxB1b	**FAIENII**A	62.5%	169–176	324	*M. musculus*	X92592
	FoxB1	**FAIENII**A	62.5%	170–178	324	*H. sapiens*	Q99853
	FoxB2	**FAIENII**G	62.5%	176–183	317	*X. laevis*	CAD31848
	FoxB2	**FAIENII**G	62.5%	267–274	428	*M. musculus*	NP032049
	FoxB2	**FAIENII**G	62.5%	266–273	425	*R. norvegicus*	109459945
	FoxB2	**FAIENII**G	62.5%	270–277	432	*H. sapiens*	61966923
C							
	FoxC	**FSVDNIM**T	75%	233–300	497	*B. floridae*	57337372
	FoxC1.1	**FSVDNIM**T	62.5%	277–284	476	*D. rerio*	AF219949
	FoxC1.2	**FSMDTIM**T	75%	254–261	433	*D. rerio*	AF219950
	FoxC1	**FSMDTIM**T	75%	275–282	470	*T. nigroviridis*	47220394
	FoxC1	**FSVDNIM**T	75%	298–305	495	*X. laevis*	80478512
	FoxC1	**FSVDNIM**T	75%	275–282	528	*G. gallus*	CAA76851
	FoxC1	**FSVDNIM**T	75%	308–315	553	*M. musculus*	AAH52011
	FoxC1	**FSVDNIM**T	75%	307–314	502	*B. taurus*	76639995
	FoxC1a	**FSVDNIM**T	75%	308–315	553	*H. sapiens*	Q12948
	FoxC1b	**FSVDNIM**T	75%	308–315	553	*H. sapiens*	AAC72915
	FoxC2	**FSVENIM**T	75%	258–265	463	*X. laevis*	47497986
	FoxC2	**FSVENIM**T	75%	244–251	445	*G. gallus*	AAC60065
	FoxC2	**FSVETIM**T	75%	269–276	494	*M. musculus*	Q61850
	FoxC2	**FSVENIM**T	75%	270–277	501	*H. sapiens*	Q99958
D							
	FoxD	**FSIESLI**G	62.5%	377–384	506	*C. savignyi*	BAB68347
	FoxD	**FSIENII**G	75%	311–318	402	*B. floridae*	AF512537
	FoxD1	**FSIDNII**G	75%	295–302	363	*D. rerio*	AAH75922
	FoxD1.1	**FSIDSII**G	62.5%	277–284	343	*D. rerio*	45501117
	FoxD1	**FSIESII**G	62.5%	294–301	345	*X. laevis*	3892202
	FoxD1	**FSIESII**G	62.5%	377–384	440	*G. gallus*	AAB08467
	FoxD1	**FSIESLI**G	62.5%	364–371	455	*R. norvegicus*	XP001057782
	FoxD1	**FSIESLI**G	62.5%	365–372	456	*M. musculus*	AAC42042
	FoxD1	**FSIESII**G	62.5%	362–369	465	*H. sapiens*	Q16676
	FoxD2	**FSIDNII**G	75%	276–283	346	*X. laevis*	CAC69867
	FoxD2	**FSIDNII**G	75%	365–372	443	*G. gallus*	AAC60064
	FoxD2	**FSIDHIM**G	62.5%	409–416	492	*M. musculus*	NP032619
	FoxD2	**FSIDHIM**G	62.5%	412–419	495	*H. sapiens*	55956928
	FoxD3	**FSIENII**G	75%	297–304	371	*D. rerio*	AAC06366
	FoxD3a	**FSIENII**G	75%	297–304	371	*X. laevis*	CAC12963
	FoxD3b	**FSIENII**G	75%	297–304	371	*X. laevis*	CAC12895
	FoxD3	**FSIENII**G	75%	319–326	394	*G. gallus*	AAC60066
	FoxD3	**FSIENII**G	75%	366–373	469	*M. musculus*	NM010425
	FoxD3	**FSIENII**G	75%	378–385	478	*H. sapiens*	NP036315
	FoxD4	**FSIESIM**Q	62.5%	324–331	408	*H. sapiens*	18959276
	FoxD4	**FTIESIM**Q	62.5%	320–327	444	*M. musculus*	6679841
	FoxD5	**FSIDSIM**A	62.5%	254–261	321	*D. rerio*	NP571345
	FoxD5a	**FSIENIM**R	62.5%	285–292	352	*X. laevis*	AAD47811
	FoxD5b	**FSIENIM**K	62.5%	285–292	353	*X. laevis*	CAB44729
	FoxD5c	**FSIENIM**G	62.5%	281–288	342	*X. laevis*	CAB44730
E							
	FoxE	**FSIENII**G	75%	207–214	393	*C. intestinalis*	BAC57420
	FoxE1	**FRINSLI**G	62.5%	202–209	354	*D. rerio*	XP696065
	FoxE1	**FRINNLI**G	62.5%	206–213	363	*T. nigroviridis*	47214250
	FoxE1	**FSINTLI**G	62.5%	231–238	379	*X. laevis*	46198238
	FoxE3	**FSIDNII**S	87.5%	269–276	422	*D. rerio*	118918391
	FoxE3	**FSIDSLI**N	62.5%	215–222	365	*X. laevis*	6642989
	FoxE3	**FSIDSLI**S	62.5%	239–246	383	*G. galus*	118094619
	FoxE3	**FRLDSLL**G	50%	195–202	288	*M. musculus*	7657098
	FoxE3	**FSVDSLV**P	50%	179–186	385	*C. familiaris*	73977761
	FoxE3	**FSVDSLV**N	50%	217–224	319	*H. sapiens*	CAI14973
	FoxE3	**FRLDSLL**G	50%	193–200	286	*R. norvegicus*	XP233428
	FoxE4	**FSIDNII**A	75%	227–234	381	*B. floridae*	18653452
G							
	FoxG1	**FSIRRML**S	62.5%	20–27	402	*B. floridae*	AF067203
	FoxG1	**FSVERLL**S	75%	286–293	402	*B. floridae*	AF067203
	FoxG1	**FSINSLV**P	62.5%	18–25	420	*D. rerio*	18858707
	FoxG1	**FSINSLM**P	62.5%	18–25	436	*X. laevis*	AAC79501
	FoxG1	**FSINSLV**P	62.5%	18–25	451	*G. gallus*	U47275
	FoxG1	**FSINSLV**P	62.5%	18–25	481	*M. musculus*	AAB42158
	FoxG1	**FSINSLV**P	62.5%	18–25	480	*R. norvegicus*	6978845
	FoxG1a	**FSINSLV**P	62.5%	18–25	469	*H. sapiens*	CAA55038
	FoxG1b	**FSINSLV**P	62.5%	18–25	477	*H. sapiens*	X74142
H							
	FoxH1	**FAIDSLL**H	50%	250–257	472	*D. rerio*	18858709
	FoxH1	**FAIDSLL**H	50%	278–285	285	*T. nigroviridis*	47223489
	FoxH1	**FMIDSLL**H	50%	271–278	518	*X. laevis*	P70056
	FoxH1	**FSIKSLL**G	62.5%	198–205	401	*R. norvegicus*	XP235454
	FoxH1	**FSIKSLL**G	62.5%	167–174	310	*B. taurus*	CAD58794
	FoxH1	**FSIKSLL**G	62.5%	198–205	401	*M. musculus*	6679845
	FoxH1	**FSIKSLL**G	62.5%	194–201	612	*H. sapiens*	41107639
I							
	FoxI1	**FSVNNLI**Y	75%	405–412	419	*D. rerio*	AAO63568
	FoxI1c	**FSVNSLI**Y	62.5%	367–374	381	*X. laevis*	CAD31849
	FoxI1c	**FTVNSLI**Y	62.5%	345–352	359	*G. gallus*	50747424
	FoxI2	**FSVNSLI**Y	62.5%	369–376	383	*D. rerio*	AAP92808
Q							
	FoxQ1	**FSIESIL**S	75%	268–275	385	*C. intestinalis*	70569660
	FoxQ1	**FSIDAIL**S	75%	226–233	324	*B. floridae*	CAH55831
	FoxQ1	**FAIDSIL**S	62.5%	177–184	383	*D. rerio*	AAH67139
	FoxQ1	**FRIDSLL**S	62.5%	276–283	383	*D. rerio*	AAH67139
	FoxQ1	**FTIDSIL**S	75%	196–203	272	*T. nigroviridis*	47220396
	FoxQ1	**FAIDSIL**S	62.5%	224–231	381	*X. laevis*	76152394
	FoxQ1	**FAIDSIL**S	62.5%	268–275	400	*M. musculus*	31560693
	FoxQ1	**FAIDSIL**S	62.5%	252–259	439	*R. norvegicus*	12408312
	FoxQ1	**FAIDSIL**R	50%	270–277	402	*H. sapiens*	8489093
	FoxQ2	**FTIDYLL**Y	62.5%	17–24	244	*D. rerio*	XP694156
	FoxQ2	**FTIDYLL**F	62.5%	20–27	210	*T. nigroviridis*	47209212
	FoxQ2	**FSIDRLV**G	62.5%	4–110	271	*B. floridae*	AY163864
L							
	FoxL1	**FSIDSIL**S	75%	284–291	363	*D. rerio*	41055835
	FoxL1	**FSIDSIL**A	62.5%	255–262	336	*M. musculus*	NP032050
	FoxL1	**FSIDSIL**A	62.5%	259–266	389	*R. norvegicus*	109508994
	FoxL1	**FSIDSIL**A	62.5%	262–269	346	*B. taurus*	61823329
	FoxL1	**FSIDSIL**A	62.5%	272–279	356	*C. familiaris*	73956953
	FoxL1	**FSIDSIL**A	62.5%	261–268	245	*H. sapiens*	22779860

^a ^The highly conserved core of the eh1-like motifs are indicated in bold.

^b ^The percent similarity between the identified Fox eh1-like motifs and the eh1 motif (FSISNILS) of the *Drosophila *engrailed homeodomain protein [7].

^c^The location of the motifs within the amino acid sequence of the individual Fox proteins.

To validate the results of the manual search for eh1-like motifs, we used the expectation-maximization algorithm in the MEME program [22]. We initially examined 18 FoxD3-related protein sequences, which contain a conserved and functional eh1 motif [21]. As predicted, the analysis identified eh1-like motifs (E-value of 10^-75^) at 18 sites corresponding to the previously described eh1 motif of FoxD3. When this approach was extended to the entire Fox family of 458 proteins, eh1-like motifs were identified at 213 sites in ten Fox subclasses (E-value of <10^-16^). The eh1-like motifs identified using the expectation-maximization algorithm corresponded to motifs identified in the manual sequence analysis, as well as to motifs previously identified in the Fox family [12,23].

To confirm the statistical significance of the match between identified eh1-like sequences and the eh1 consensus, a hidden Markov model (HMM) was constructed [24] for the eh1 motif of FoxD3 (eh1 FD3). This model of the eh1 motif was used to search the SWISS protein database and a summary of the results of the eh1 FD3 HHM analysis is shown in Table 4. A total of 49,363 matches with the eh1 motif were identified, and 647 matches were to proteins that are members of transcription factor families. The mean log-odds score for all transcriptional proteins was 9.07, whereas non-transcriptional proteins scored at 6.87. Among transcriptional proteins, Fox family proteins resulted in the strongest matches with the eh1 motif, with a mean log-odds scores of 14.34. The motifs were identified in 9 subclasses of the Fox protein family which included A, B, C, D, E, G, H, L and Q (the FoxI subclass is not represented in the current SWISS protein database). The search also identified a significant number of high scoring matches (mean log-odds score of 11.61) for homeodomain-containing proteins of the para-Hox cluster [25], but the score for other non-Fox, non-para-Hox transcriptional proteins was low (7.72). The results of the HMM analysis strongly supports the conclusion that eh1-like motifs are present within proteins of the Fox family at high frequency when compared with most transcriptional protein families and non-transcriptional proteins.

**Table 4:** Descriptive statistics of the Meta-MEME search of SWISS protein database^a ^using a hidden Markov model of the FoxD3 eh1-like motif.

**Protein class^b^**	**Log-Odds^c ^Mean (SD)**	**Log-Odds Minimum**	**Log-Odds Maximum**	**Hits^d^**
Non-Transcription	6.87 (2.24)	1.49	24.43	48716
Fox	14.34 (5.65)	5.97	29.23	54
Para-Hox	11.61 (4.83)	3.64	23.45	155
Other Transcription	7.72 (2.67)	3.48	17.42	318
All Transcription	9.07 (4.28)	3.48	29.23	647

^a ^SWISS protein database integrated in the Meta-MEME software package (version 3.2).

^b ^Protein classes were defined by the presence of a conserved DNA-binding domain for transcriptional proteins, or by the absence of a DNA-binding domain for non-transcriptional proteins. Non-Transcription, proteins that are not members of defined families of transcriptional proteins; Fox, Fox family proteins; Para-Hox, para-Hox class of homeodomain proteins; Other Transcription, transcriptional proteins excluding Fox and para-Hox proteins; All Transcription, all transcriptional proteins.

^c ^Log-odds score is the ratio of a sequence score with respect to the foreground model versus the sequence score with respect to the background model. The log-odds score is the logarithm of an odds score in base 2. SD, standard deviation.

^d ^Hits are positions in the background sequence that align with a motif model.

To evaluate the statistical significance of the eh1-like motif identification results obtained by HMM, logistic regression analysis was performed. Analysis of the log-odds scores for the transcriptional protein and non-transcriptional protein classes indicated that the association of eh1-like motifs with transcriptional proteins had high statistical significance (p < 2 × 10^-9^). Furthermore, analysis of the log-odds scores for the Fox family transcriptional proteins and other transcriptional protein classes were analyzed, the association of higher log-odds scores with Fox proteins was found to have high statistical significance (p < 2 × 10^-9^). The results strongly support the conclusion that eh1 motifs are present in members of the Fox family at high frequency, and suggest that the eh1 motif contributes to the transcriptional function of many Fox family proteins.

For most of the Fox proteins analyzed, a single eh1-like motif was located C-terminal to the WHD (Fox subclasses A, B, C, D, E, H, I, L and Q). Two similar eh1-like motifs are present in the zebrafish FoxQ1 protein, with both C-terminal to the WHD. Interestingly, the *C*. *elegans *FoxD and sea urchin, amphioxus and zebrafish FoxQ2 proteins contain N-terminal eh1-like motifs, whereas a C-terminal motif location is found for the other FoxD and FoxQ orthologs. All FoxG proteins contain an eh1-like motif N-terminal to the WHD, and in sea urchin and amphioxus FoxG proteins a second eh1-like motif is located C-terminal to the WHD. The vertebrate FoxG proteins contain a C-terminal sequence that appears to be a remnant of an eh1 motif that lacks the conserved phenylalanine. Eh1-like motifs were identified in Fox proteins in several early divergent species. These included sponge (phylum Porifera) FoxD, hydra and sea anemone (phylum Cnidaria) FoxA, and comb jelly (phylum Ctenophora) FoxG. The presence of eh1 motifs in Fox proteins of these phyla suggests an ancient appearance of this motif in the Fox gene family and therefore, a functional interaction with Groucho-related corepressors early in the evolution of the Fox gene family.

### Loss of eh1-like motifs within Fox gene subclasses

**Figure 1:**
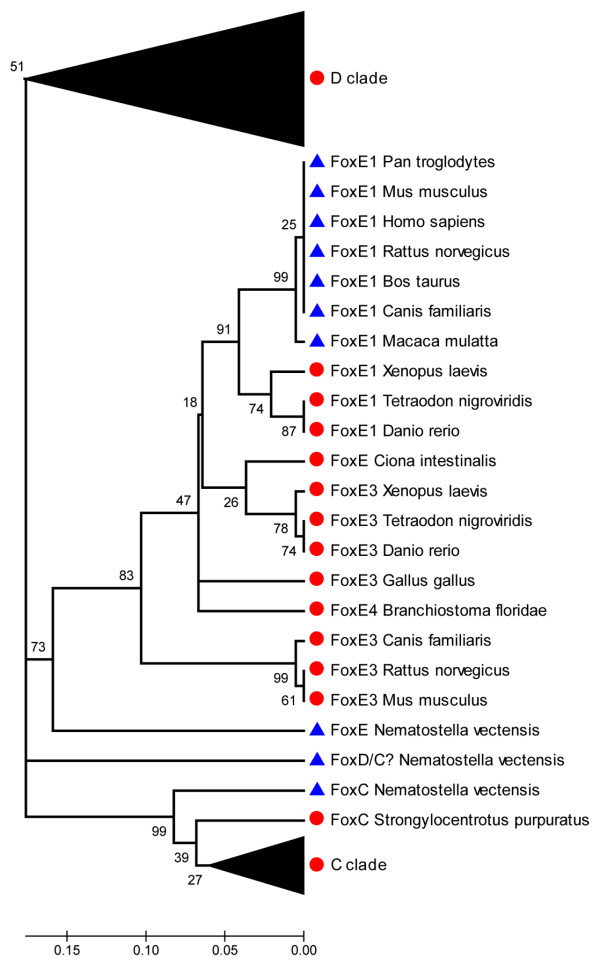
A phylogenetic tree for proteins of the FoxE subclass and the FoxC and FoxD outgroups. A neighbor-joining method was used to construct the tree topology and bootstrapping values are shown at each branch point (percentage of 1000 bootstrap samples) using the MEGA 3.1 software. Gaps were deleted in pairwise comparisons. The distance scale below the tree represents the number of substitutions per site. The C and D families are collapsed for better illustration. Protein sequences that lack a recognizable eh1-like motif are represented by blue triangles. Proteins and subclasses that contain an eh1-like motif are represented by red circles.

Our sequence analysis indicates incomplete distribution of the motif within certain Fox subclasses, suggesting the loss of the motif in a subset of Fox proteins. A striking example of the loss of the eh1-like motif is observed within the FoxE subclass for FoxE1 proteins. Sequence analysis of FoxE subclass proteins did not identify a recognizable eh1 motif in seven mammalian FoxE1 proteins, whereas FoxE1 proteins of fish and amphibia, and nine other FoxE proteins contained the motif. To assess the inheritance and loss of the eh1 motif during the evolution of FoxE proteins, a phylogenetic tree for the FoxE subclass and the FoxC and FoxD outgroups was constructed using a neighbor-joining method (Figure 1). The topology of the phylogenetic tree (bootstrap value 91%) indicates a close relatedness of the fish, amphibian, and mammalian FoxE1 proteins, which suggests a common ancestry. Therefore it is reasonable to infer that the ancestral FoxE1 protein contained the motif, and the loss of the eh1 motif occurred in the mammalian lineage or ancestors of the mammalian phyla in the course of evolution. All other members of the FoxE subclass, including the amphioxus and tunicate proteins, as well as mammalian FoxE3 proteins, contained the motif. This suggests that most likely an ancestral FoxE protein contained the motif before the separation and expansion of the FoxE subclass, and this idea is supported by the presence of the motif in nearly all members of the FoxC and FoxD outgroups.

It should be noted that a cnidarian FoxE-related protein lacks the eh1 motif, and this may be viewed as inconsistent with the presence of the eh1 motif in the ancestral FoxE protein. However, phylogenetic analysis indicates a distant relatedness of this cnidarian protein to the FoxE subclass, arguing for different origins. Similarly, the motif is not detected in the *N. vectensis *FoxD- and FoxC-related proteins, which also appear to have undergone significant sequence divergence. The motif is present in cnidarian FoxA and FoxB proteins, as well as the FoxC- and FoxD-related (Fox1) proteins of the sponge *S. domuncula *[see Additional files 1 and 2], suggesting that ancestral precursors for these subclasses contained the motif, whereas the motif was likely lost in a subset of more divergent cnidarian Fox proteins.

No eh1-like motif is detected in the tunicate FoxH-like proteins, whereas nearly all vertebrate FoxH proteins contain the motif. The absence of the eh1 motif in the tunicate FoxH proteins suggests a divergence and loss of this motif in the hemichordate lineage. However, it is also possible that the ancestral FoxH protein did not contain an eh1 motif and that the motif was recruited in the vertebrate lineage. Interestingly, a *Xenopus *FoxH1 paralog, FoxH3, also lacks the eh1 motif present in other vertebrate FoxH orthologs, again suggesting a loss of the motif, perhaps due to functional specialization [see Additional files 1 and 2].

### Characteristics of eh1-like motifs in Fox family proteins

**Figure 2:**
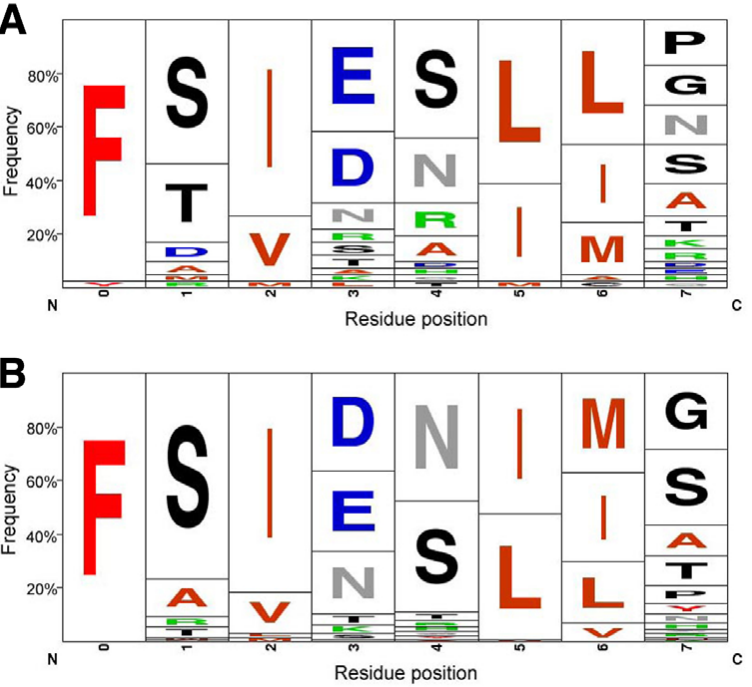
The diagrams summarize the amino acid compositions of the eh1-like motifs identified in Fox proteins. The amino acid usage frequency of eh1-like motifs identified in invertebrate (A) and vertebrate (B) Fox proteins. The diagrams were generated with the WebLogo program [44].

For the eh1-like motifs identified, the amino acid frequency at each position of the motif was determined to better define the characteristics of the motif in invertebrate and vertebrate members of the Fox gene family (Figure 2). For this frequency analysis, each position in the motif is identified as 0 to 7 in an N-terminal to C-terminal order. Although this analysis includes Fox proteins of evolutionary distant organisms, similar residue usage is observed at most positions. Overall, the identified motifs are characterized by the predominance of hydrophobic residues. The aromatic residue, phenylalanine, is absolutely conserved (100%) at position 0 of the identified motifs in vertebrates and in nearly all invertebrates. The hydrophobic core of the motif (positions 2, 5 and 6) is characterized by the frequent presence of branched hydrophobic residues such as isoleucine, leucine, methionine, and, less frequently, valine. For both vertebrates and invertebrates, isoleucine is highly represented at position 2 (75%), and leucine and isoleucine appear at similar frequencies (40–60%) at positions 5 and 6  in both invertebrates and vertebrates. Serine is highly represented at position 1 (75%) in vertebrate Fox proteins, whereas serine (55%) and threonine (30%) predominate at this position in invertebrates. Although positions 3 and 4 are variable, there is a strong bias for negatively charged residues at position 3 and the uncharged polar residues serine and asparagine at position 4. Position 7 of the eh1-like motifs is most variable, with glycine, alanine and serine residues often present. It should be noted that within individual Fox subclasses, residue identity at each position is more highly conserved, reflecting the evolutionary relatedness of the proteins in each subclass, as well as the conservation of subclass-specific functional and structural properties of the motifs [see Additional files 2 and 3].

The conservation of multiple hydrophobic residues in the eh1 motif is favorable for the formation of α-helices, and suggests that the eh1-like motifs identified in Fox family proteins have the potential to adopt a hydrophobic α-helical structure. To predict structural characteristics of the motifs, several algorithms (DSC, PHD, MLRC) were used to calculate the propensity of secondary structure formation [26-28]. For several Fox proteins of each subclass, regions containing the eh1-like motif were analyzed for predicted secondary structure. The results obtained using multiple algorithms predict a high likelihood of α-helical structure in the region of the eh1-like motif for the majority of Fox proteins examined. The highest scores for α-helical propensity were obtained for the eh1-like motifs present in FoxB, FoxE and FoxQ proteins, and α-helical structure was also predicted for FoxD, FoxA, FoxC and FoxL proteins, albeit with lower propensity scores [see Additional file 4 and data not shown].

**Figure 3:**
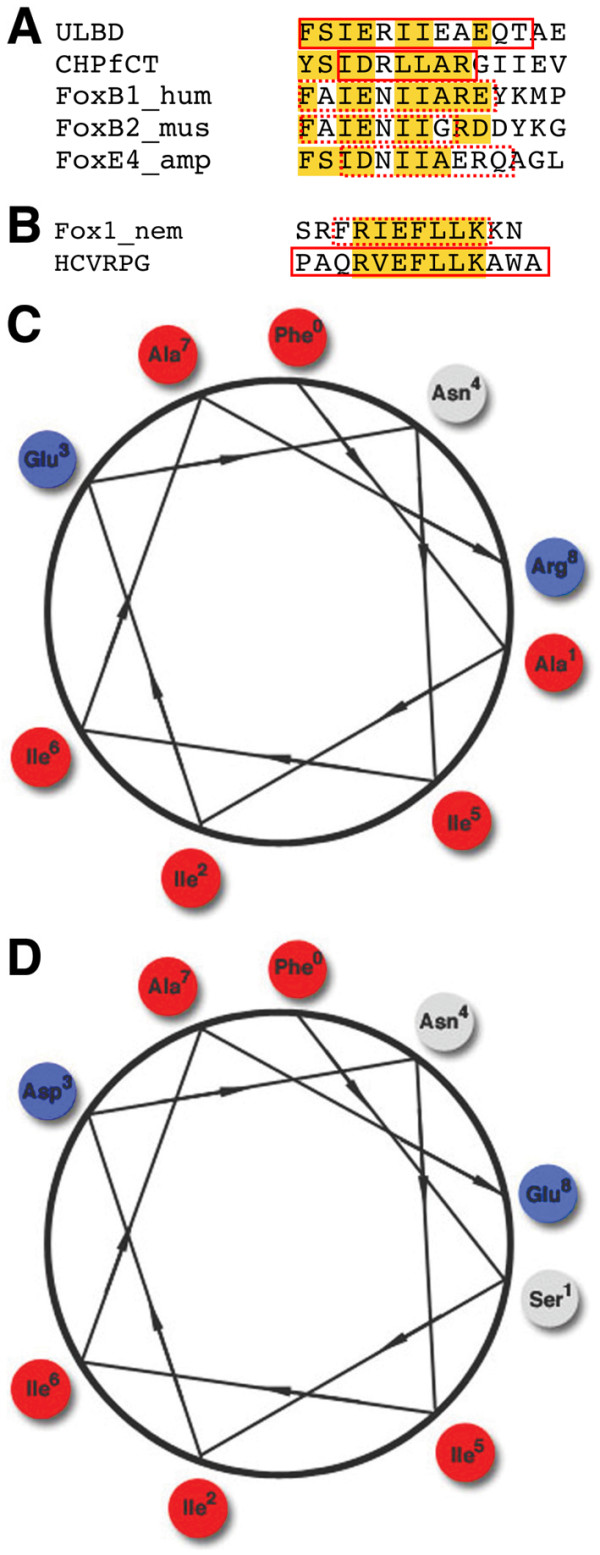
(A) Multiple sequence alignments of the α-helical region of an ultraspiracle ligand binding domain from *Drosophila *(ULBD), α-helix of a conserved hypothetical protein from *C. tepidum *(CHPfCT), and the eh1 motifs of human FoxB1, murine FoxB2 and amphioxus FoxE4 proteins, which have a high likelihood of α-helix formation. (B) Sequence alignment for the α-helical region of the Hepatitis C Virus RNA Polymerase Genotype 2a (HCVRPG) and the eh1 motif of the cnidarian Fox1 protein. The defined α-helices are represented as red solid boxes and predicted α-helices are shown as red dotted boxes. Amino acid similarities are shown in yellow. hum, Human; mus, Mouse; amp, amphioxus; nem, Sea Anemone. Wheel models of the eh1-like motifs of *Xenopus *FoxB1 (C) and amphioxus FoxE4 (D) form an amphipathic α-helical structure. Hydrophobic residues on the wheel are shown in the red, hydrophilic residues are shown in the blue, and non-charged residues are shown in the gray.

In BLAST searches, the eh1-like motifs of several Fox proteins, including FoxB and FoxE proteins, show similarity to the hydrophobic regions of several membrane proteins, including the α-helical regions of the *Chlorobium tepidum *segregation and condensation protein B (CHPfCT, AAM71720), *Pseudomonas aeruginosa *probable transcriptional regulator Pa0477 (2ESND), and *Drosophila *ultraspiracle ligand-binding domain (ULBD, 1HG4F) (Figure 3A and data not shown). A BLAST search for sequences related to the *N. vectensis *Fox1 eh1-like motif identified the α-helical region of Hepatitis C RNA Polymerase (1YVZA) as the only related sequence (Figure 3B). The ability of eh1-like sequences in proteins unrelated to the Fox family to form α-helical structure supports the prediction of α-helical structure for the eh1-like motifs identified in Fox proteins.

Helical wheel analysis of the predicted α-helical regions of the eh1-like motifs revealed an amphipathicity for a majority of the identified motifs. As an example of this analysis, the helical wheel models of the eh1-like motifs of FoxB1 and FoxE4 (Figure 3C,D) display a predicted amphipathicity of the α-helical structure. For both eh1-like motifs, a hydrophobic surface is formed by Isoleucine residues at positions 2, 5 and 6 of the predicted α-helix. The eh1-like motifs of a subset of FoxB1, FoxB2, FoxH1 and FoxQ1 proteins contain an additional hydrophobic residue (Alanine or Methionine) at position 1 that extends the hydrophobic surface of the predicted α-helix (Figure 3C and data not shown). Opposite the hydrophobic surface of the predicted α-helix is a surface consisting predominantly of hydrophilic and non-charged residues (Figure 3C,D and data not shown). Thus, the majority of the eh1-like motifs identified in Fox proteins have a predicted amphipathic α-helical structure. The validity of the predicted eh1 structure is strongly supported by a recent crystallographic study showing that the Goosecoid eh1 motif forms a short amphipathic α-helix when bound to the WD domain of TLE1 [29].

### Positional distribution of C-terminal eh1-like motifs

**Figure 4:**
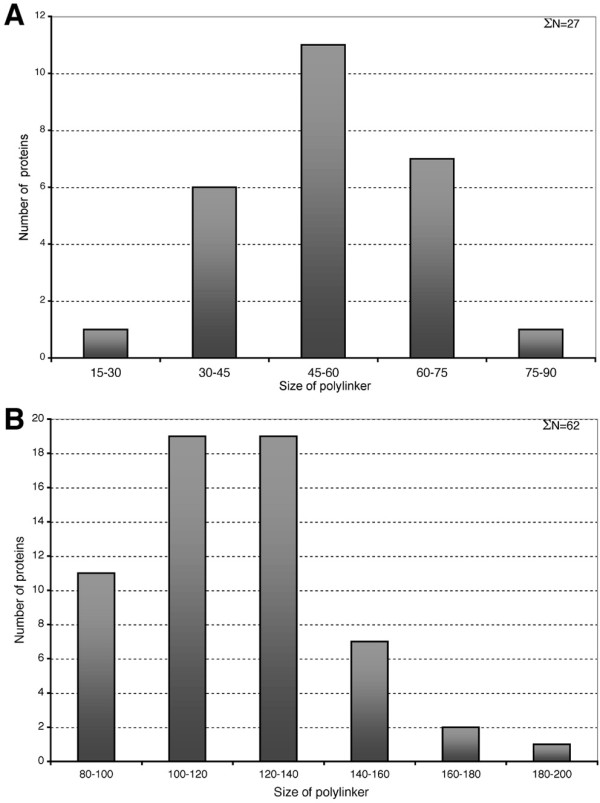
The positional distribution of the C-terminal eh1-like motifs in Fox proteins of the B, E, H and Q subclasses (A) and the A D, C and I subclasses (B). Size of polylinker represents the distance between the first residue of the eh1 motif and the conserved C-terminal residue of the winged helix DNA-binding domain.

The eh1-like motifs identified in the Fox family were further analyzed for motif position within individual Fox proteins. Given that nearly all of the eh1-like motifs identified in the Fox family are positioned C-terminal to the WHD, we limited the analysis to C-terminal motifs. To assess the variation in motif position within the C-terminus of Fox proteins, the positional distribution of the eh1-like motifs relative to the WHD was examined. A substantial variation in the relative positions of the C-terminal eh1-like motifs and the WHDs was found, with an interval ranging from 30–180 residues (Figure 4). A detailed analysis of the positional distribution of these domains in 89 Fox protein sequences revealed two groups, C-proximal and C-distal, defined by maximum interval occurrence between the two domains. For the C-proximal eh1 motifs the maximum interval occurrence is 45–60 residues with a median value of 58 residues (Figure 4A). For the C-distal motifs the maximum interval occurrence is 100–140 residues with a median value of 120 residues (Figure 4B).

**Figure 5:**
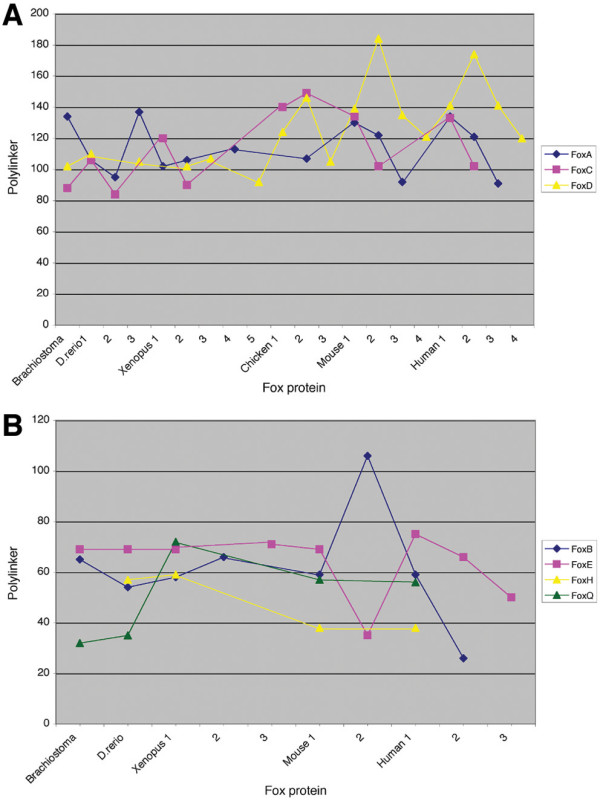
Positional fluctuations of eh1-like motifs in the ortholog and paralog groups of vertebrate Fox proteins. (A) Positional fluctuations of the eh1-like motifs of the ortholog and paralog groups of the A, C and D subclasses. (B) Positional fluctuations of the eh1-like motifs of the ortholog and paralog groups of the B, E, H and Q subclasses. Polylinker represents the distance between the first residue of the eh1-like motif and the conserved C-terminal residue of the winged helix DNA-binding domain. The paralog groups within a Fox subclass are indicated on the *x*-axis.

Positional variation of the C-terminal eh1-like motifs was also examined within Fox ortholog and paralog groups for eight subclasses. This analysis was limited to chordate Fox proteins as non-chordates lack many Fox subclasses. Proteins of Fox subclasses B, E, H and Q contain C-proximal motifs, whereas C-distal motifs are present in Fox subclasses A, C, D and I. The positional distribution of the motifs in the ortholog groups is shown in Figure 5. The analysis indicates that the position of eh1-like motifs is conserved within individual Fox protein subclasses across species, but not across subclasses within individual species. This conservation of motif position within each subclass is consistent with the existence of a common ancestral gene for the Fox genes comprising an individual subclass [17], but may also reflect a functional constraint that maintains the position of the eh1 motif. Exceptions to the conservation of motif position are observed for the FoxD and FoxQ subclasses, and for orthologs of FoxA3, FoxC1, and FoxH1. For the FoxD subclass, a shift of motif position towards the C-terminus is observed for chick, mouse and human proteins, when compared to amphixous, zebrafish and *Xenopus *(Figure 5A). A C-terminal shift is also observed for the eh1 motifs of *Xenopus*, mouse and human FoxQ proteins, compared to amphioxus and zebrafish (Figure 5B). Similarly, for FoxC1 proteins, the eh1 motif of the chick and mammalian orthologs is shifted C-terminally in comparison to the zebrafish and *Xenopus *orthologs. In contrast, the eh1 motif of mammalian FoxH1 proteins is shifted N-terminally, closer to the WHD, in comparison to the zebrafish and *Xenopus *proteins.

For each case where eh1 motif position is not conserved, the shift in motif position correlates with changes in the size of the coding region C-terminal to the WHD. For example, sequence alignment of FoxD subclass proteins reveals the presence of polyalanine, polyglycine and polyproline repeats in the mammalian proteins that are absent in FoxD proteins of lower vertebrates (data not shown). On the other hand, mammalian FoxH1 proteins lack sequences C-terminal to the WHD that are present in the *Xenopus *and zebrafish orthologs (data not shown). Thus, insertion or deletion of sequences within the C-terminal domain of these mammalian Fox proteins is likely responsible for the shift of eh1 motif position.

## Discussion

In this study, we have identified the presence of eh1-like Groucho interaction motifs in ten subclasses of the Fox family of transcriptional regulators by systematically analyzing 458 protein sequences of nineteen Fox subclasses. The analysis shows a widespread distribution of eh1-like motifs within the Fox protein family. The presence of the motif was identified in Fox subclasses, A, B, C, D, E, G, H, I, L and Q, and no eh1-like motif was detected in proteins of the F, J, K, M, N, O, P, R and S subclasses. The majority of the eh1-like motifs identified were located C-terminal to the WHD, including proteins of nine Fox subclasses (A, B, C, D, E, H, I, L and Q). Only the FoxG subclass proteins contained eh1-like motifs N-terminal in the WHD. For Fox proteins containing C-terminal eh1-like motifs, the position of the motif relative to the WHD defined a C-proximal group with motifs 45–60 residues from the WHD (Fox subclasses B, E, H and Q) and a C-distal group with motifs 100–140 residues from the WHD (Fox subclasses A, C, D and I). The presence of eh1 motifs in more than 50% of Fox family proteins was in marked contrast to other protein families, including both transcriptional and non-transcriptional proteins (Table 4 and data not shown).

The prevalence of eh1-like motifs in the Fox family suggests that Groucho corepressors directly interact with many Fox proteins to mediate transcriptional repression activity or to inhibit the activation function of other regulatory domains. In a number of cases the functional importance of the identified eh1-like motifs is confirmed by the presence of the motifs within defined transcriptional repression domains and by the ability to mediate direct binding to Groucho proteins. The eh1 motifs are present in the C-terminal repression domains of mouse and chick FoxD3 [30,31], and *Xenopus *FoxD5 [32], as well as the C-terminal transcriptional inhibitory domain of mouse FoxC1 [33]. Furthermore, the eh1 motifs mediate a functional and direct interaction with Groucho corepressors in mouse FoxA2 [19], *Drosophila *FoxG/sloppy-paired-1 [20], mouse FoxG1 [34], and *Xenopus *FoxD3 [21] and FoxH1 (SY and DSK, unpublished). These results confirm the importance of eh1 motifs in Fox family proteins, and suggest that the eh1-like motifs identified in this study may mediate a previously unappreciated interaction of Groucho corepressors with many Fox proteins.

Secondary structure analysis of the eh1-like motifs indicates that a majority of the identified motifs are highly likely to form an α-helical structure. In support of this secondary structure prediction, a number of the eh1-like motifs exhibit sequence similarity to regions of unrelated proteins with known α-helical structure. In addition, the eh1-like motifs exhibit amphipathicity, which argues in favor of α-helix formation by the motifs. Structural studies of a number of transcriptional regulators have demonstrated the importance of amphipathic α-helices in binding to transcriptional coregulators. The p53 tumor suppressor binds to the transcriptional coactivator, MDM2, via a 13 amino acid motif. Structural studies have shown that the MDM2 interaction motif of p53 forms an amphipathic α-helix that binds to MDM2 through hydrophobic interactions [35]. In addition, NRSF/REST binds to the Sin3 corepressor via several short amphipathic or hydrophobic α-helices [3]. Therefore, the predicted amphipathic α-helical structure of the eh1 motifs is likely an essential feature for direct, high-affinity binding of Fox proteins to Groucho corepressors. This conclusion is strongly corroborated by recent structural studies showing that the eh1 motif present in the human Goosecoid protein forms a short amphipathic α-helix when bound to the WD domain of the Groucho family protein TLE1 [29]. In general, these observations support the idea that diverse families of transcriptional regulators utilize distinct conserved motifs, which adopt a common amphipathic α-helical structure, as adaptors for the physical interaction with transcriptional coregulators.

Eh1-like motifs were identified in Fox proteins of the most evolutionary ancient organisms, including marine sponge (porifera), comb jelly (ctenophora) and sea anemone (cnidaria). The presence of the eh1-like motif in Fox proteins of these organisms likely reflects the presence of the eh1-Groucho interaction functional module early in evolutionary history. Eh1-like motifs are also present in other transcriptional regulators of the sponge, including the Barx/Bsh1 (AAQ24371) and a paraHox-related homeodomain protein (CAD37941). Consistent with the presence of eh1-like motifs in transcriptional regulatory proteins of early divergent species, a Groucho gene (CN626783) has been identified in the cnidarian Hydra. These data suggest an ancient origin for eh1 motif-dependent recruitment of Groucho corepressors, a protein interaction that may have been established as early as the porifera.

An intriguing question raised by these analyses is the origins of the eh1 motifs in the Fox gene family. The motifs identified in all Fox subclasses, except for the FoxG subclass, are positioned C-terminal to the WHD. The occurrence of the eh1-like motif N-terminal to the WHD in the FoxG subclass and FoxQ2 suggests that the N-terminal motif may have arisen independent of the C-terminal motif. In addition, two eh1-like motifs, positioned N-terminal and C-terminal to the WHD, were identified in the sea urchin and amphioxus FoxG1 proteins. The presence of two motifs in distinct regions of a subset of FoxG1 orthologs is consistent with independent origins for the C-terminal and N-terminal eh1 motifs. Given the small size of the eh1 motif (8 residues), it is possible that the motif arose multiple times in the Fox family. Therefore, the formation of new eh1-like motifs through the accumulation of missense mutations offers a convergent mechanism for multiple independent appearances of the motif in the Fox family. Alternatively, the Fox genes may have acquired the motif via a non-homologous recombination event that introduced a repression module containing an eh1-like motif. Such a scenario could involve the incorporation of a new exon encoding the repression module. However, since a majority of the Fox family genes lack introns, this mechanism would require intron loss subsequent to incorporation of the eh1-encoding exon.

An apparent loss of eh1 motifs was observed in a subset of FoxD, FoxE, and FoxH proteins. Our analysis indicates that the loss of the motif occurred in a subset of mammalian Fox proteins and we speculate that the motif loss provided a new functional modification for these proteins that was evolutionarily beneficial. Since the presence of an eh1 motif likely mediates a functional interaction with Groucho corepressors, the loss of the motif may represent an alteration of both transcriptional activity and regulatory function for individual Fox proteins. For example, while FoxH1 proteins can function as transcriptional activators or repressors by recruitment of Smad coactivators or Groucho corepressors [36,37] (SY and DSK, unpublished), it is predicted that FoxH3 functions exclusively as an activator in association with Smad coactivators [38]. Thus, the eh1 motif may play an important role in the evolution of the Fox gene family by providing a basis for the evolutionary modification of Fox protein function.

## Conclusion

The identification of eh1-like motifs in many members of the Fox gene family provides an important insight into the potential transcriptional activity of Fox family proteins, and provides a foundation for the study of eh1 motif function in the Fox family. Biochemical and transcriptional studies will now be necessary to determine if the identified eh1-like motifs mediate a direct physical interaction with Groucho corepressors to confer transcriptional repression activity. Building on our motif analyses, ongoing functional studies should yield a more comprehensive understanding of the evolution, domain organization, and transcriptional activity of the Fox gene family.

## Methods

### Manual sequence analysis

The Fox gene family is subdivided into nineteen subclasses on the basis of homology within the winged helix DNA-binding domain [15], and at the time of this study the nineteen subclasses comprised 458 sequences. To identify eh1-like motifs, we used the eh1 consensus sequence F^0^S/A^+1^Φ^+2^X^+3^X^+4^Φ^+5^Φ^+6^X^+7 ^(Φ, branched hydrophobic residues; X, non-polar or charged residues), which has been generated based on the published data. Yeast and metazoan Fox protein sequences present in the SWISS-PROT and NCBI databases were analyzed. To identify the presence of an eh1-like motif in protein sequences of the nineteen subclasses, we performed PSI-BLAST searches of the non-redundant databases with inclusion threshold (E-value) of 0.01 using members of each Fox subclass as a query. In parallel, the sequences of all subclasses were retrieved from the NCBI database and multiple protein alignments were constructed for each subclass using the CLUSTAL W algorithm in the software package MacVector 7.2.2. Regions that were conserved within either the N-terminal or C-terminal regions of at least two species were examined for a minimum of 50% similarity to the eh1 consensus. Taken together these searches allowed for the identification of conserved sequences matching the eh1 consensus in ten Fox subclasses.

### Expectation-maximization and hidden Markov model analyses

The expectation-maximization algorithm of the MEME program **(**Multiple Em for Motif Elicitation, version 3.5.4) [22,39] was used to analyze 458 proteins of the Fox family for the presence of eh1-like motifs. The search parameters used were 20–30 motifs per a run and a motif size of 8–10 amino acid residues.

An eh1 motif position-specific probability matrix was generated for a set of FoxD3 protein sequences using MEME, and this matrix was used to construct a hidden Markov model for eh1-like motifs using the Meta-MEME program (Motif-based hidden Markov modeling of biological sequences, version 3.2) [24,40]. The SWISS protein database was searched with the FoxD3 eh1-like motif model using an E-value threshold of <10^4 ^for reported sequences.

Logistic regression analysis was performed to determine whether there was a statistically significant correlation between the results of the hidden Markov model analysis (log-odds scores) and all transcriptional proteins or Fox family proteins specifically. The dependent variable in the logistic regression analysis is the dummy variable (*y*), which is equal to 1 when a transcriptional protein is present and 0 otherwise. The independent variable is the score (*x*). The estimated logistic regression equation is: y^=ea+bx1+ea+bx
 MathType@MTEF@5@5@+=feaafiart1ev1aaatCvAUfKttLearuWrP9MDH5MBPbIqV92AaeXatLxBI9gBaebbnrfifHhDYfgasaacH8akY=wiFfYdH8Gipec8Eeeu0xXdbba9frFj0=OqFfea0dXdd9vqai=hGuQ8kuc9pgc9s8qqaq=dirpe0xb9q8qiLsFr0=vr0=vr0dc8meaabaqaciaacaGaaeqabaqabeGadaaakeaacuWG5bqEgaqcaiabg2da9maalaaabaGaemyzau2aaWbaaSqabeaacqWGHbqycqGHRaWkcqWGIbGycqWG4baEaaaakeaacqaIXaqmcqGHRaWkcqWGLbqzdaahaaWcbeqaaiabdggaHjabgUcaRiabdkgaIjabdIha4baaaaaaaa@3E0F@, where *x *is the score and y^
 MathType@MTEF@5@5@+=feaafiart1ev1aaatCvAUfKttLearuWrP9MDH5MBPbIqV92AaeXatLxBI9gBaebbnrfifHhDYfgasaacH8akY=wiFfYdH8Gipec8Eeeu0xXdbba9frFj0=OqFfea0dXdd9vqai=hGuQ8kuc9pgc9s8qqaq=dirpe0xb9q8qiLsFr0=vr0=vr0dc8meaabaqaciaacaGaaeqabaqabeGadaaakeaacuWG5bqEgaqcaaaa@2E37@ is an estimate of the probability that *y *= 1 or that the transcription factor is present given the score.

### Phylogenic analysis of Fox proteins

A phylogenic tree for the FoxE subclass was generated based on the winged-helix DNA-binding domain sequences (100 residues) for FoxC, FoxD and FoxE subclass proteins. Multiple sequence alignments were constructed using Clustal W [41] and these sequences were converted into a cladogram using MEGA 3.1 [42]. Distances were calculated with Poisson correction, and a neighbor-joining method was used to construct the tree topology with bootstrap analysis of 1000 samples.

### Secondary structure analysis

For secondary structure predictions, the C-terminal or N-terminal domain of selected Fox proteins of each subclass was subjected to analysis using algorithms that predict secondary structure with accuracy in the range of 0.67–0.7. The prediction algorithm is available at the Network Protein Sequence Analysis website [43]. The source code of the combiner can be obtained on request for academic use. In addition, software written by M.L. (unpublished) was used to predict the secondary structure of Fox protein sequences. This helix prediction algorithm is based on all high-resolution structures available, with the scoring function comparing homology of the sequences to known helical structures.

## Authors' contributions

SY initiated these studies and was involved in all aspects of the design, execution and interpretation of these studies, as well as the writing of the manuscript. AV participated in the motif search and statistical analyses, and contributed to the writing of the manuscript. SS and ML contributed to the secondary structure analysis and amphipathic modeling. DSK contributed to the design and interpretation of these studies, data presentation and writing of the manuscript. All authors read and approved the final manuscript.

## Supplementary Material

Additional file 1 Phylogenetic Tree of the Fox Gene Family Indicating the Occurrence of eh1 Motifs. A phylogenetic tree of the entire Fox gene family indicating which individual proteins contain an eh1-like motif. Click here for file

Additional file 2 Legends for Additional Files 1 and 3. Description of data presented in Additional Files 1 and 3. Click here for file

Additional file 3 The amino acid composition of eh1-like motifs identified in individual Fox protein subclasses. Diagrams representing the amino acid composition of the eh1-like motifs identified in each Fox family subclass of invertebrate and vertebrate organisms. Click here for file

Additional file 4 Propensity for α-helix formation for eh1-like motifs in selected Fox proteins. An analysis of the propensity for α-helix formation at the position of individual residues within the eh1-like motifs of selected Fox family proteins. Click here for file
