# Supplementary figures and images for: Prevalence of the EH1 Groucho interaction motif in the metazoan Fox family of transcriptional regulators
Source: BMC Genomics. 2007 Jun 28;8:201. doi: 10.1186/1471-2164-8-201 (PMC1939712; doi:10.1186/1471-2164-8-201)

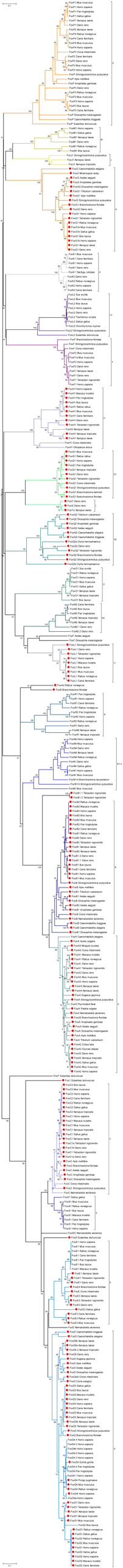

Supplement: Additional file 1 — Phylogenetic Tree of the Fox Gene Family Indicating the Occurrence of eh1 Motifs. A phylogenetic tree of the entire Fox gene family indicating which individual proteins contain an eh1-like motif. [file 1471-2164-8-201-S1.jpeg]

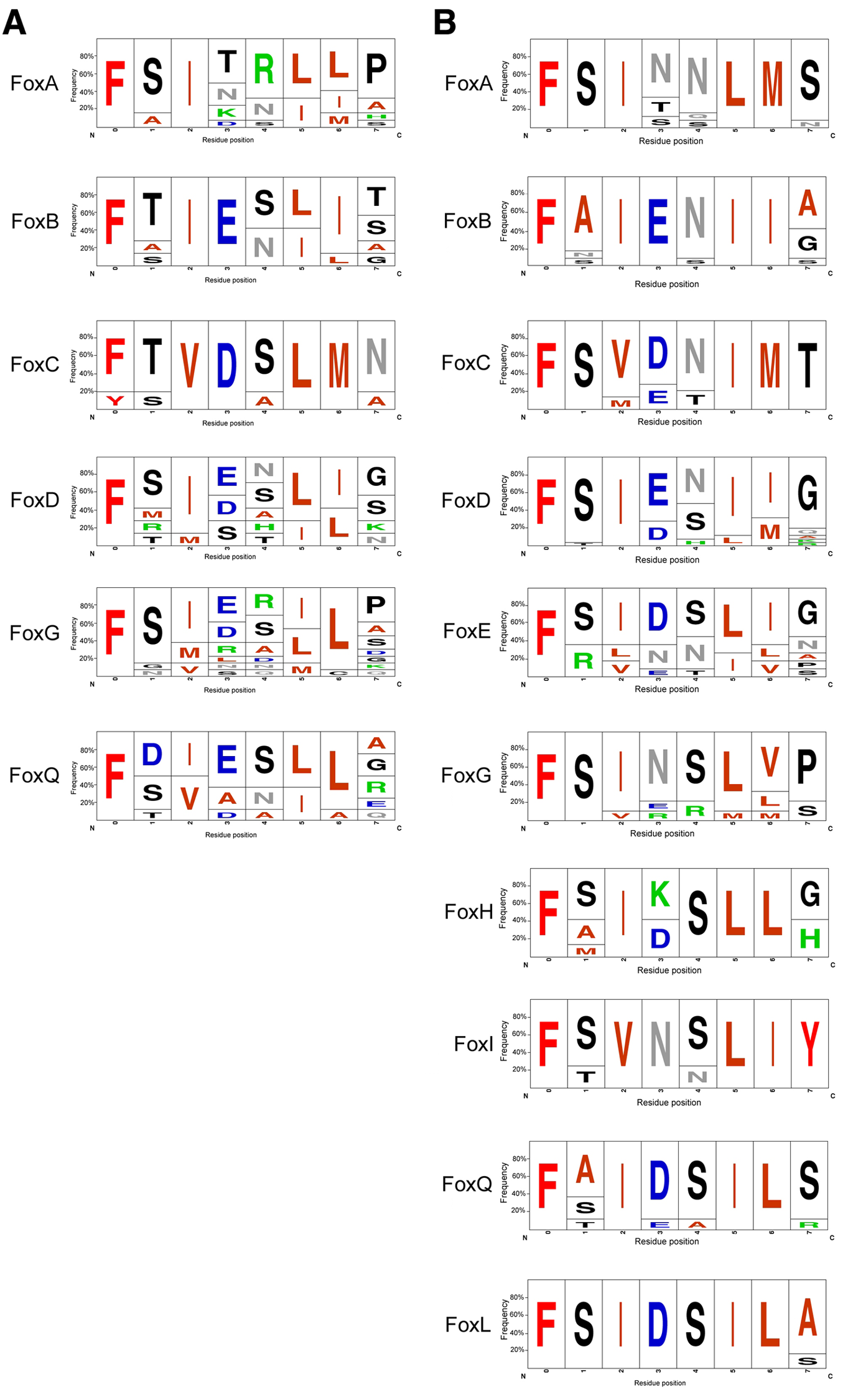

Supplement: Additional file 3 — The amino acid composition of eh1-like motifs identified in individual Fox protein subclasses. Diagrams representing the amino acid composition of the eh1-like motifs identified in each Fox family subclass of invertebrate and vertebrate organisms. [file 1471-2164-8-201-S3.tiff]
