# Supplementary material for: Prevalence of the EH1 Groucho interaction motif in the metazoan Fox family of transcriptional regulators
Source: BMC Genomics. 2007 Jun 28;8:201. doi: 10.1186/1471-2164-8-201 (PMC1939712; doi:10.1186/1471-2164-8-201)
Supplement: Additional file 2 — Legends for Additional Files 1 and 3. Description of data presented in Additional Files 1 and 3. [file 1471-2164-8-201-S2.doc]

**Figure Legends for Additional Files 1 and 3**

Additional File 1. Phylogenetic relationships of the Fox protein family. A neighbor-joining method was used to construct the tree topology and bootstrapping values are shown at each branch point (percentage of 1000 bootstrap samples) using software MEGA 3.1. Proteins that contain an identified eh1-like motif are indicated with red circles. The distance scale represents the number of substitutions per site. For the FoxI and FoxJ subclasses only a subset of known proteins is included.

Additional File 3. The diagrams summarize the amino acid composition of eh1–like motifs identified in individual Fox protein subclasses. The amino acid usage frequency of eh1-like motifs identified in invertebrate (A) and vertebrate (B) Fox proteins. The color scheme represents hydrophobic and aromatic residues (FYWILVA) in red, acidic residues (ED) in blue, alkaline residues (KR) in green, non-charged residues (QN) in gray, and other residues in black. The diagrams were generated using the WebLogo program [44].
