# Supplementary material for: Prevalence of the EH1 Groucho interaction motif in the metazoan Fox family of transcriptional regulators
Source: BMC Genomics. 2007 Jun 28;8:201. doi: 10.1186/1471-2164-8-201 (PMC1939712; doi:10.1186/1471-2164-8-201)
Supplement: Additional file 4 — Propensity for α-helix formation for eh1-like motifs in selected Fox proteins. An analysis of the propensity for α-helix formation at the position of individual residues within the eh1-like motifs of selected Fox family proteins. [file 1471-2164-8-201-S4.doc]

Additional File 4. Propensity for α-helix formation for eh1-like motifs in selected Fox proteins.

| **Protein** | **Propensity of α-helix formation**^a^ | | | | | | | | | | | **Score**^b^ |
| --- | --- | --- | --- | --- | --- | --- | --- | --- | --- | --- | --- | --- |
|  | 0 | +1 | +2 | +3 | +4 | +5 | +6 | +7 | +8 | +9 | +10 |  |
| FoxB1 | F | A | I | E | N | I | I | A | R | E | Y |  |
|  | 8 | 9 | 9 | 9 | 9 | 9 | 9 | 9 | 9 | 8 | 5 | 93 |
|  | H | H | H | H | H | H | H | H | H | H | H |  |
|  |  |  |  |  |  |  |  |  |  |  |  |  |
| FoxE4 | F | S | I | D | N | I | I | A | E | R | Q |  |
|  | 6 | 6 | 7 | 8 | 9 | 9 | 9 | 9 | 9 | 9 | 8 | 89 |
|  | H | H | H | H | H | H | H | H | H | H | H |  |
|  |  |  |  |  |  |  |  |  |  |  |  |  |
| FoxQ1 | F | A | I | D | S | I | L | R | K | P | F |  |
|  | 9 | 9 | 9 | 8 | 8 | 9 | 8 | 6 | 2 | 2 | 2 | 72 |
|  | H | H | H | H | H | H | H | H |  |  |  |  |
|  |  |  |  |  |  |  |  |  |  |  |  |  |
| FoxD4 | F | T | I | E | S | I | M | Q | G | V | T |  |
|  | 1 | 2 | 5 | 8 | 8 | 9 | 8 | 6 | 3 | 2 | 1 | 53 |
|  |  |  | H | H | H | H | H | H |  |  |  |  |
|  |  |  |  |  |  |  |  |  |  |  |  |  |
| FoxA | F | S | I | N | N | L | M | S | S | S | E |  |
|  | 1 | 3 | 5 | 7 | 7 | 8 | 7 | 7 | 1 | 1 | 2 | 49 |
|  |  |  | H | H | H | H | H | H |  |  |  |  |
|  |  |  |  |  |  |  |  |  |  |  |  |  |
| FoxC1 | F | S | V | D | N | I | M | T | S | L | R |  |
|  | 1 | 0 | 1 | 5 | 7 | 8 | 7 | 5 | 5 | 3 | 2 | 44 |
|  |  |  |  | H | H | H | H | H | H |  |  |  |
|  |  |  |  |  |  |  |  |  |  |  |  |  |
| FoxD5 | F | S | I | E | N | I | M | R | K | P | K |  |
|  | 1 | 2 | 5 | 6 | 7 | 8 | 7 | 2 | 0 | 0 | 0 | 38 |
|  |  |  | H | H | H | H | H |  |  |  |  |  |
|  |  |  |  |  |  |  |  |  |  |  |  |  |
| FoxL1 | F | S | I | D | S | I | L | S | K | K | E |  |
|  | 2 | 2 | 5 | 6 | 8 | 8 | 6 | 3 | 1 | 0 | 0 | 39 |
|  |  |  | H | H | H | H | H |  |  |  |  |  |
|  |  |  |  |  |  |  |  |  |  |  |  |  |
| FoxD1 | F | S | I | E | S | I | I | G | G | S | L |  |
|  | 2 | 2 | 5 | 6 | 7 | 6 | 4 | 1 | 1 | 1 | 1 | 36 |
|  |  |  | H | H | H | H | H |  |  |  |  |  |

^a^ The propensity of α-helix formation at the position an individual residues in the eh1-like motifs (calculation based on the algorithm described in [28]).

^b^ The score represents the sum of the individual propensity values at each residues of the eh1-like motifs.
